# Negligible contribution of body density to in-water vertical jump performance in elite male water polo players

**DOI:** 10.1371/journal.pone.0311273

**Published:** 2025-01-07

**Authors:** Bence Balázs, Balázs Sebesi, Alexandra Cselkó, Márk Váczi

**Affiliations:** 1 Institute of Sport Sciences and Physical Education, Faculty of Sciences, University of Pécs, Pécs, Hungary; 2 Doctoral School of Biology and Sport Biology, Faculty of Sciences, University of Pécs, Pécs, Hungary; 3 Pecsi Sport Nonprofit Zrt., Pécs, Hungary; 4 Doctoral School of Health Sciences, Faculty of Health Sciences, University of Pécs, Pécs, Hungary; 5 Hungarian School Sport Federation, Budapest, Hungary; University of L’Aquila Department of Clinical Sciences and Applied Biotechnology: Universita degli Studi dell’Aquila Dipartimento di Scienze Cliniche Applicate e Biotecnologiche, ITALY

## Abstract

The aim of this study was to investigate the associations among in-water vertical jump and various dry-land physical measures by taking the law of Archimedes into consideration, and by normalizing the dry-land measures both to body density and body mass. Seventeen elite water polo players from Hungarian first league were tested for dry-land counter movement and squat jump mechanical impulse, isometric squat force, and in-water vertical jump height. Body density was estimated by anthropometric measurements. Body density alone did not influence in-water vertical jump height. Both the impulse of counter movement jump normalized to body mass (9.42±0.86 N·s/kg) and impulse of counter movement jump normalized to body density (773.92±109.68 N·s/kg/g/cm^3^) correlated with the in water vertical jump height (69.37±6.12 cm) (p≤0.05), but the magnitudes of the correlation coefficients 0.49 vs. 0.50 were not statistically different (p = 0.480, z = 0.04). Neither the impulse of squat jump normalized to body mass (7.07±0.59 N·s/kg) nor impulse of squat jump normalized to body density (577.87±89.16 N·s/kg/g/cm^3^) correlated with the in water vertical jump height (both p>0.05). The correlation between the force of the maximum voluntary isometric contraction normalized to body density (1052.13±244.65 N/kg/g/cm^3^) and the in water vertical jump height only approached the level of significance (p = 0.077). We concluded that dry-land reactive strength determines the ability to jump out of water, but players’ body density does not seem to contribute to jump height.

## Introduction

The physical demand in water polo has dramatically increased in the last decades. Game rules have been continuously changed with the aim to make this sport faster and more spectacular, challenging the players’ conditional skills, and launching more sophisticated training systems involving both in-water and dry-land physical preparation [1]. As an adaptation to the increased training volume, water polo players today represent higher levels of condition and leaner body than in the last century [2, 3].

Lower limb strength plays an important role in various water polo play elements [3] and its dry-land development is encouraged [4]. Lower limb strength enables the player to perform quick game actions in water either with or without the ball. According to game analyses [5], a special treading foot movement called "eggbeater kick" is used on an average of 240 times in a water polo match to change direction, start, fight body-to-body, block, and shoot. Eggbeater kick requires a rotational and treading movement by the legs and small cyclical and symmetrical movements by the hands, which generate a downward force and an upward body movement in the water [6].

The in-water vertical jump (IWVJ) test (i. e. jumping as high as possible from the water) has been developed and validated to assess players’ in-water lower limb function needed in the aforementioned egg-beater kick maneuver. The measurement of IWVJ performance has drawn a considerable attention by researchers as it is often associated with swim speed and swim agility [7]. In addition, studies have shown that IWVJ performance is a predictor of throwing velocity in elite female players [8, 9]. Conditioning specialists often incorporate dry-land resistance exercises into the training program with a hope of transferring strength into in-water lower limb function [10]. The question whether dry-land physical performance predicts in-water performance has been in the focus of researchers. The associations among dry-land strength measures and IWVJ performance have been tested in a few studies [11–13], founding no correlation between the dry-land vertical jump and IWVJ performance. The conclusion is that the ability to jump vertically in water depends a large extent on the level and in-game position of the player, rather than dry-land vertical jump performance [11, 12]. Lower body maximum strength has also been considered to contribute to certain water polo play elements. The throwing velocity of a water polo player, for example, is influenced by the maximum strength of the lower body, which means that the player must exert force to propel the body out of the water. However, for a lower-level player, strength alone is not enough to jump high from the water, a properly developed egg-beater kick is also required [8].

An important limitation in the aforementioned studies is that researchers did not consider fluid mechanics and did not control for the players’ body density when they tested IWVJ. Namely, the position of an object immersed in fluid is determined by the magnitude of the gravity force and the buoyancy that opposes gravity force. As an object with greater body mass and body volume is exposed to greater gravity force and buoyancy, respectively, the density of the object (i. e. mass per unit volume) determines whether it sinks or floats. Considering the principle above, it is assumed that buoyancy (at least in part) promotes IWVJ performance. Therefore, correlation would be expected between IWVJ performance and the various dry-land physical measures, if the physical measures were normalized to the player’s body density.

In the present study, we approached the aforementioned problem by taking the effects of buoyancy and gravity force into account, and normalizing the dry-land physical performance values of elite male water-polo players both to their body mass and body density for testing the following hypotheses: (i) IWVJ height correlates both with dry-land vertical jump performance and lower limb strength, and that (ii) these correlations become stronger when dry-land vertical jump performance and lower limb strength are normalized to body density versus body mass.

## Materials and methods

### Participants

Seventeen elite water polo players (age: 20.5±2.83 years, body mass: 88.7±10.5 kg, height: 189.8±5.05 cm) were recruited from 1 to 12 September 2020 from the same club team. The team currently played in the Hungarian first division league and placed 9^th^ in the previous season. Exclusion criteria were current injuries such as sprains or strains in the lower limb or trunk as well as suffering from illness, but all players were deemed to be in good health and were able to participate in the experiment. Before any testing, a written informed consent was signed to participate in the study which informed the subjects about the conduct of the study, conditions of participation, risks and cancellation options. The protocol was approved by the Regional Ethics Committee (8197 –PTE 2020).

### Experimental protocol

Players attended two test sessions with one day in between. In test session 1, body density was estimated as well as dry-land countermovement (CMJ) and squat jump (SJ) mechanical impulse, and maximal voluntary isometric contraction (MVIC) force in a squat position were measured. Before any testing, players warmed-up by cycling for 5 minutes on a cycle ergometer at a self-selected speed, and by stretching. In test session 2, IWVJ height was measured. Subjects warmed up on dry- land using dynamic stretching exercises. In-water warm up involved 200 meters swimming in self-selected stroke and dynamic eggbeater kick drills. The experiment was conducted at the end of the pre-season preparation, when players trained an average of 17 hours (12 hours in water, 5 hours dry land) per week, arranged in 13 training sessions. The goal during the pre-experiment training period was to develop endurance, strength, and tactics. In-water training involved interval training, agility, and practicing power-play situations. Dry-land training involved circuit resistance training and stretching.

### Body density estimation

Body mass (BM) was measured using Multi-Frequency Bioelectrical Impedance (InBody 770, Biospace Co.,). Body density (BD) was estimated from the biceps, triceps, subscapular, and suprailiac skinfolds (Lange caliper, Model SH5020, Saehan Corporation, Korea), using the equation by Durnin és Wormersley [14]: BD = 1.1631 –(0.0632 · L), where BD = Body Density, and L = Log of the sum of skinfolds.

### CMJ and SJ tests

Players stood on a force plate (Tenzi, Pilisvorosvár, Hungary) with both legs. Three bilateral CMJs and SJs were performed with 1 min rest between trials. It was required to place the hands on the hips to avoid arm-swing contribution during the jumps, and players were instructed to jump as high as possible. In SJs, further instruction was given to avoid any preliminary countermovement. When countermovement was noticed visually on the force-time curve, the trial was repeated. During jumps, vertical ground reaction force was measured with respect to time (sampling frequency: 420 Hz), and from the force-time curve the propulsive impulse was calculated as follows:

$$\begin{array}{c} t2\\ J^{\to }=\int \;F\;dt\\ t1 \end{array}$$

where J⃗ = propulsive impulse, F→ = force acting on the body over a time interval from t1 to t2. Values were then normalized both to body mass (I_CMJ/BM_, I_SJ/BM_) and to body density (I_CMJ/BD_, I_SJ/BD_) of the player. The best CMJs and SJs were considered for statistics.

### Isometric squat test

Players stood on the same force plate as in the jump tests, under an Olympic barbell attached to a squat stand. The isometric squat test was performed with knee angle of 90°. As a warm-up, three submaximal trials were performed at 70, 80, and 90% of self-estimated maximum intensity. Players were instructed to “push as hard as possible” to produce a maximum contraction for five seconds. The force achieved in the best trial (F_MVIC_) was normalized to the participants’ body mass (F_MVIC/BM_) and body density (F_MVIC/BD)_) and taken into account in the data analysis.

### In-water vertical jump test

**Fig 1:**
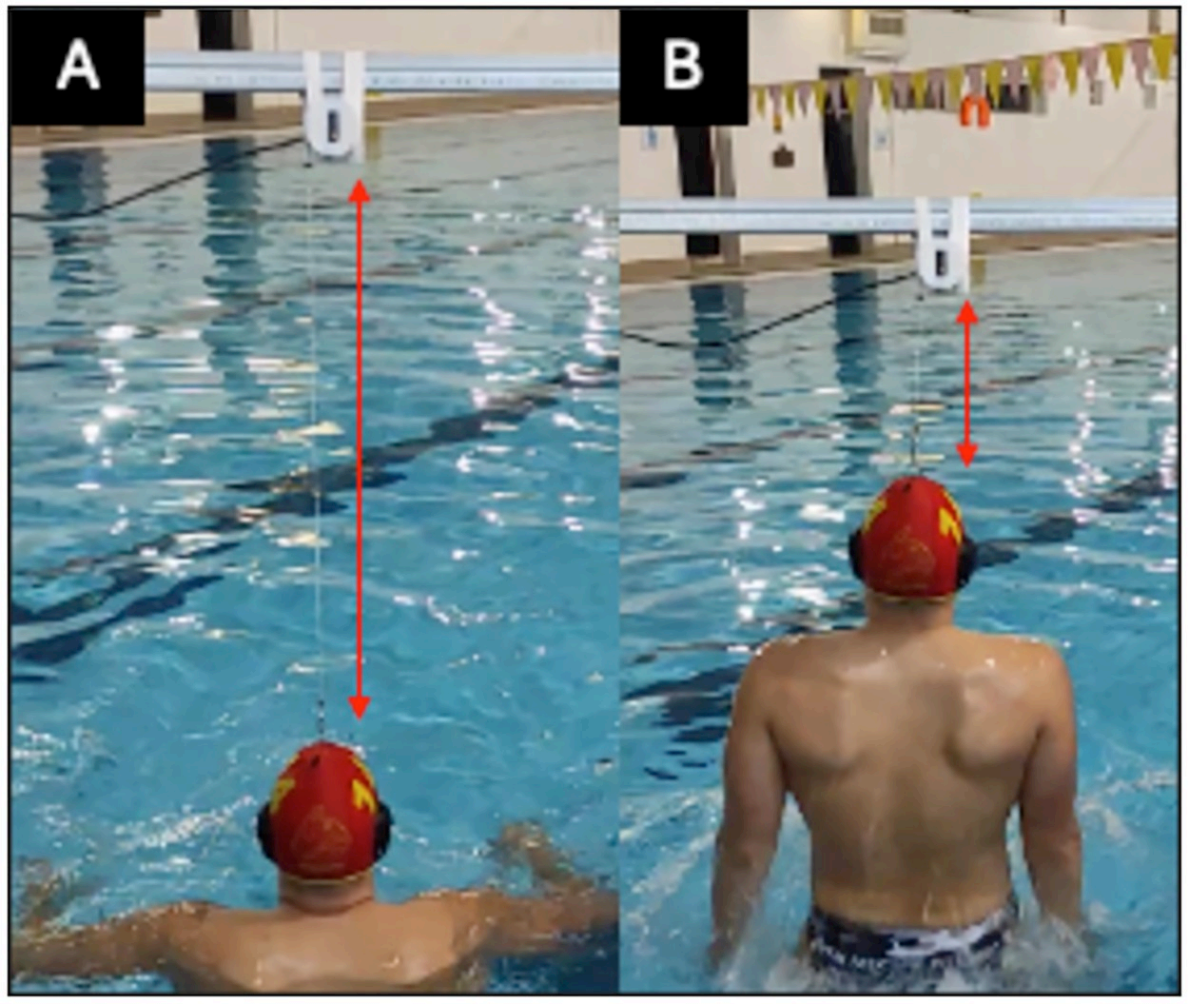
Setup of the in-water vertical jump (IWVJ) test. A: starting position, the red line indicates the length of the string at the start position. B: maximum height achieved; the red line indicates the length of the string during the in-water vertical jump.

The IWVJ height was measured using a linear encoder (Chronojump-Boscosystem, Barcelona, Spain). The setup is shown in Fig 1. The linear encoder was fixed to an aluminum rod, which crossed the water at 1m height. The string of the linear encoder was hooked into the player’s cap. The IWVJ was initiated from the fundamental floating position, exactly under the device, when the acromion and the water surface were in level. To ensure the vertical position of the string before the jump, vertical bars were used as references outside the pool. Players performed three attempts with arm boost, with 2 min rest between trials. The linear encoder recorded the jump height, which was immediately displayed by the software (Chronojump-Boscosystem, Barcelona, Spain).

### Statistical analysis

Mean, standard deviation, range, and 95% confidence interval values are presented for all variables. All data were normally distributed. To test hypothesis 1, Pearson correlation coefficients were used to determine the relationship among all measured and calculated variables (SPSS, Version 17.0; Chicago, IL), involving both the body mass- and body density-normalized values obtained from the jump and MVIC tests. To test hypothesis 2, we analyzed the differences among the obtained correlation coefficients by using Fisher’s r to z transformation technique [15]. Intra-class correlation coefficients (ICC) were computed to test absolute agreement type test-retest reliabilities in the performance measures. The ICCs (and the 95% confidence intervals) were as follows: 0.98 in CMJ impulse (95% CI: 0.96–0.99), 0.97 in SJ impulse (95% CI: 0.94–0.99), 0.96 in MVIC force (95%CI: 0.93–0.98), 0.93 in IWVJ height (95% CI: 0.86–0.97). The ICCs for skinfold measurements (biceps, triceps, subscapular, and suprailiac) were all above 0.99.

## Results

Table 1 shows the players’ anthropometric characteristics as well as the dry-land and the in-water performance measures.

**Table 1:** Mean, standard deviation (SD), range, and 95% confidence interval (CI) values for the measured and calculated variables obtained in two separate laboratory test sessions in elite male water polo players (n = 17).

Variables	Mean	SD	Range	CI (95%)
**Body Mass (kg)**	88.72	10.47	70.4–112.3	88.72 ± 4.97
**Body Density (g/cm3)**	1.05	0.007	1.03–1.06	1.05 ± 0.003
**CMJ Impulse (N·s)/BM)**	9.42	0.86	8.07–10.7	9.42 ± 0.4
**SJ Impulse (N·s)/BM)**	7.07	0.59	5.97–8.18	7.07 ± 0.28
**IWVJ height (cm)**	69.37	6.12	53.8–81.5	69.37 ± 2.9
**MVIC force (N)**	1107.05	258.2	623–1557	1107.05 ± 122.73
**SJ impulse (N·s/BD)**	577.87	89.16	402.2–747.7	577.87 ± 42.38
**CMJ impulse (N·s/BD)**	773.92	109.68	539–983.9	773.92 ± 52.13
**MVIC force (N/BM)**	12.65	3.15	5.5–17.6	12.65 ± 1.49
**MVIC force (N/BD)**	1052.13	244.65	593.1–1498.5	1052.13 ± 116.29

CMJ: Counter movement jump, SJ: Squat jump, IWVJ: In-water vertical jump, MVIC: Maximal voluntary isometric contraction, BM: Body mass, BD: Body Density

**Fig 2:**
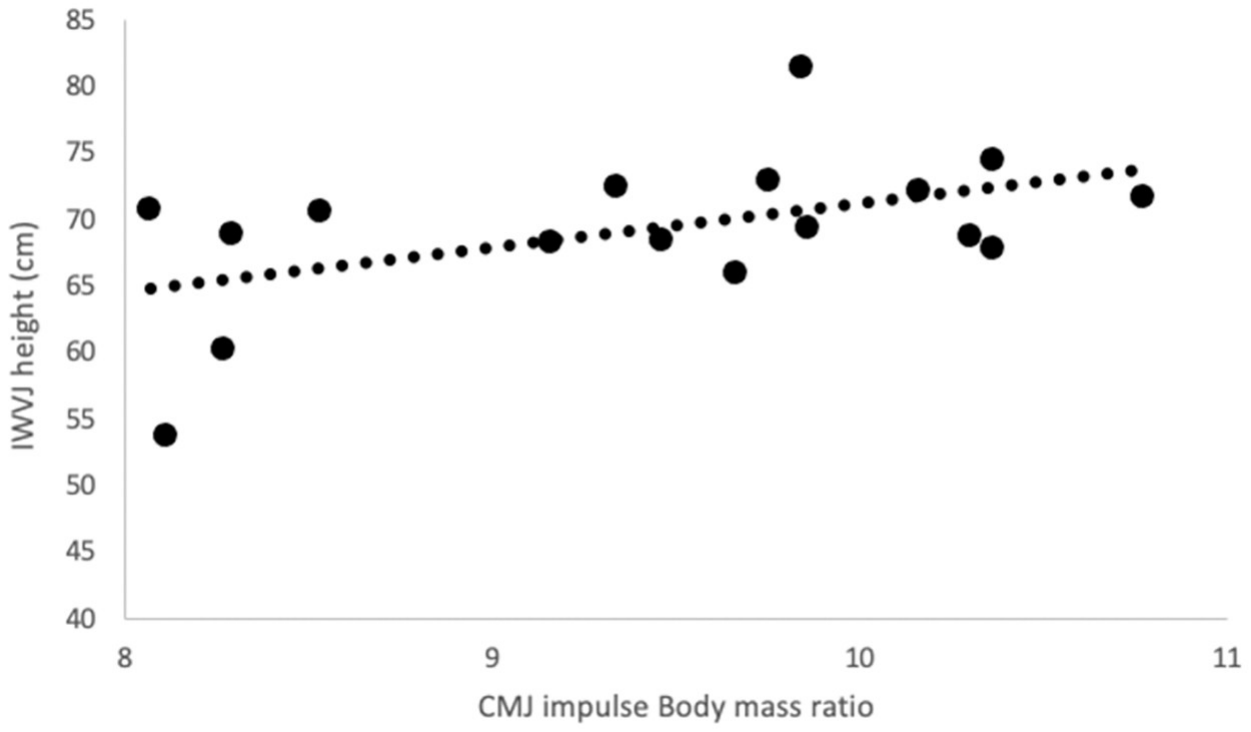
Correlation between IWVJ height and I_CMJ/BM_.

**Fig 3:**
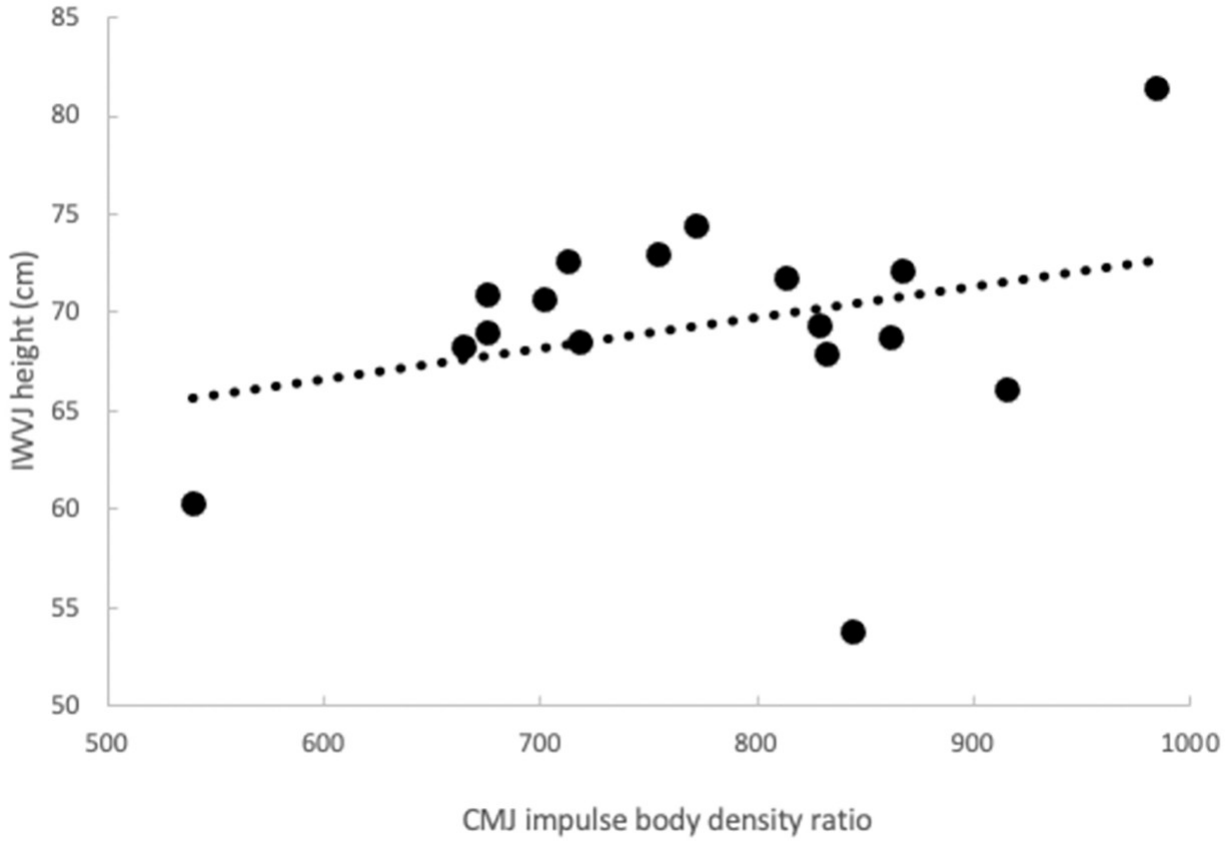
Correlation between IWVJ height and I_CMJ/BD_.

Correlation coefficients among the measured and calculated variables are presented in Table 2. Both I_CMJ/BM_ and I_CMJ/BD_ correlated with IWVJ height (both p ≤ 0.05, statistical power: 0.60, and 0.58, respectively). The plots for these correlations are presented in Figs 2 and 3, respectively. Interestingly, neither I_SJ/BD_ nor I_SJ/BM_ ratios correlated with IWVJ height (p > 0.05). The correlations between F_MVIC/BD_ and IWVJ height only approached the level of significance (p = 0.077).

**Table 2:** Correlation coefficients among the measured and calculated variables (r), *Significant at p<0.05 (n = 17).

	**BM**	**BD**	**I**_**CMJ/BD**_	**I**_**CMJ/BM**_	**I**_**SJ/BD**_	**I**_**SJ/BM**_	**F**_**MVIC/BD**_	**F**_**MVIC/BM**_
**BM**								
**BD**	-0.33							
**I**_**CMJ/BD**_	0.72	-0.31						
**I**_**CMJ/BM**_	-0.12	0.02	0.59					
**I**_**SJ/BD**_	0.84	-0.39	0.78*	0.19				
**I**_**SJ/BM**_	0.17	-0.16	0.44	0.51*	0.66			
**F**_**MVIC/BD**_	-0.06	0.16	0.21	0.34	-0.20	-0.27		
**F**_**MVIC/BM**_	-0.50	0.37	-0.19	0.29	-0.56	-0.34	0.88	
**IWVJ height**	-0.12	-0.37	0.49*	0.50*	-0.01	0.12	0.45	0.33

BM = Body mass, BD = Body density, I_CMJ/BD_ = Mechanical impulse during countermovement jump normalized to body density, I_CMJ/BM_ = mechanical impulse during countermovement jump normalized to body mass, I_SJ/BM_ = Mechanical impulse during squat jump normalized to body mass, I_SJ/BD_ = Mechanical impulse during squat jump normalized to body density, IWVJ = In-water vertical jump, F_MVIC/BD_ = Maximum voluntary isometric contraction force normalized to body density, F_MVIC/BM_ = Maximum voluntary isometric contraction force normalized to body mass

The Fisher’s r to z transformation test revealed that the magnitudes of correlations were not different either between *I*_*CMJ/BM*_ *and IWVJ height* or between *I*_*CMJ/BD*_ and *IWVJ height (z = -0*.*04)*. We found strong correlation between I_CMJ/BD_ and I_SJ/BD_ (p = 0.039).

## Discussion

In the present investigation we introduced a novel methodological approach to investigate the associations among performances in various dry-land tasks and IWVJ height in male elite water-polo players, i.e. dry-land performance measures were expressed with the consideration of players’ body density. The main finding is that though CMJ impulse contributed to IWVJ height (hypothesis 1), the correlation was not stronger when CMJ impulse was normalized to body density vs. body mass (hypothesis 2). SJ impulse and isometric squat force did not correlate with IWVJ height (hypothesis 1).

Our methodological approach was derived from the fact that when an object is immersed into fluid, the magnitudes of the two opposing forces, gravity and buoyancy, will determine whether the object floats or sinks. Given, therefore, that body mass and volume determine the aforementioned forces, we hypothesized that their ratio, i.e. body density, would influence IWVJ height. The correlation between body density and swim performance has been studied among swimmers but data is contradictory, showing either correlation [16] or no correlation [17]. In contrast with swimming movement, however, water-polo players often perform push-offs in a vertical direction, identical to the action of buoyancy. Still, we found no direct effect of body density alone on IWVJ height. We further investigated whether SJ and CMJ impulse normalized either to body mass or body density influence IWVJ height and found significant correlation only in the case of CMJ impulse (both body mass- and body density-normalized). SJ and CMJ are commonly used vertical jump tests to measure athletes’ explosive and reactive strength, respectively, in ground-contact sports (e.g. soccer, basketball, handball, track and field athletics, volleyball, etc.) [2]. Testing CMJ in water-polo players has become of interest since 2005 [4, 10, 12, 13], due to the fact that water-polo players target strength development through dry-land preparation, and, therefore, dry-land performance could predict in-water performance. These previous studies, however, failed to support the hypothesis that dry-land CMJ and IWVJ performances are related. In contrast, in the present study, we found significant correlation between IWVJ height and the body-mass-normalized CMJ impulse *(Table 2)*, suggesting that lower limb reactive strength contributes to jump ability in water. The discrepancy between our and the previous data can perhaps be explained with the different CMJ testing methodologies. Most of the previous studies used the Sargent test (a reach test, when one arm is extended overhead to reach to maximum height at jump) both dry-land and in water. In our study, the linear encoder attached to the players’ cap allowed both arms to remain in water when the push-off was performed, and we obtained high reliability for both CMJ and IWVJ tests (ICC = 0.98 and 0.93, respectively).

Interestingly, we found no correlation between SJ impulse and IWVJ height. The SJ test is used for the evaluation of explosive strength in dry-land sports. While during the eccentric phase of a CMJ muscle elastic energy is stored, which can then be re-utilized during the concentric phase [18], the SJ test is performed from a stationary squat position allowing much less elastic energy storage. In-water movements such as push-offs, eggbeater-kicks, vertical jumps, hip abductions involve concentric only muscle actions, and the possibility of elastic energy storage and its re-utilization is excluded. Therefore, we expected that SJ performance would be correlated with IWVJ height, although this correlation only approached the level of significance (p = 0.07). Test-retest reliability could not be a reason for the lack of correlation as we found an ICC of 0.95. A possible explanation could be that players had not done any SJ-type exercises during their dry-land preparation programs previously, and probably they were unable to activate their leg muscles sufficiently in this test. It is also possible that players applied non-optimal knee angles from which the SJ was initiated, or applied knee angles different form which the IWVJ was performed. As neither myoelectric activity nor joint kinematics was measured, this remains a limitation in the present study.

In our study, we used an isometric bilateral squat test for evaluating maximum strength, and we found that neither body mass- nor body density-normalized squat force correlated with IWVJ. Isometric squat force is often measured in dry-land sports as it contributes to agility, sprint speed and jump height [19], when it is normalized to body mass, commonly named as relative strength. Isometric squat strength does not seem to be a contributor to IWVJ, and, because IWVJ is performed with a rapid concentric action, perhaps using concentric dynamic dry-land strength tests with various joint angular velocities would be more appropriate. The isometric squat can only be used to assess changes in certain joint angles and body positions, which may not directly reflect the ability to perform a multi-joint task [20–22].

As mentioned earlier, an important limitation in previous studies investigating the association among dry-land and in-water performance measures in water polo players is that the law of Archimedes was ignored. We assumed that a player with higher absolute strength and smaller body density (ratio of body mass and body volume) would produce better IWVJ height as smaller density favors buoyancy against gravity, supporting the upward movement in water. Therefore, beside body mass, we also considered body density and expressed the dry-land performance measures relative to body density. Despite this speculation, we found that though CMJ impulse normalized to body density correlated with IWVJ height, this correlation was statistically not stronger than the correlation between body mass-normalized CMJ impulse and IWVJ height. Furthermore, we found the same in the case of SJ impulse and isometric squat force, rejecting our second hypothesis. Though the support of buoyancy applies to every object immersed in fluid, it is possible that the small range of body density obtained in our group of players (1.03–1.06 g/cm^3^) did not allow to prove that dry-land performance with the consideration of body density would correlate stronger with IWVJ height compared to when body density is ignored. Using the law of Archimedes, we calculated that (supposed that a player’s volume increases but his body weight does not change) a decrease of 0.03 g/cm^3^ in body density (i.e. the difference between the lower and upper value in our data line, Table 1) would reveal only 79.1 N increase in buoyancy, and this additional support in in-water upward movement could be negligible considering that the opposing force of gravity was as high as ~ 700 to 1100 N acting on our players (see Table 1), and that players had far to exceed this force in order produce ~70 cm IWVJ height. Certainly, the above calculation on the magnitude of bouyancy would only be true if players were fully immersed in water. However, players were immersed in water to the level of the shoulder acromions, and as they performed the IWVJ, trunk and arms became gradually uncovered by water, reducing the magnitude of buoyancy. Therefore, the ratio of buoyancy and gravity force continuously changed during IWVJ, which we were unable to control. Measuring body-segment specific body density along with video analysis during IWVJ would be important to better understand the contribution of buoyancy. Another important limitation is that we were unable to control under-water boost technique during IWVJ. The water polo boost technique is a complicated 3d movement (see introduction), which can influence the efficiency of the jump, and its investigation requires highly precise underwater motion analysis system. Training history and level of our players, however, suggest perhaps small inter-individual differences in boost technique, still contribution of technique vs. strength to IWVJ height should be further studied. Furthermore, the IWVJ introduced in our study does not perfectly replicate any water polo movement as an in-water jump without raising a hand is not used in real game situations. Finally, while the IWVJ tests had high reliability, the power of its correlations with other variables were below 0.8, increasing the probability of type 2 error. Still, future water polo studies can use the current data to compute effect size and power in IWVJ tests.

In conclusion, we introduced a novel methodological approach for investigating the associations among dry-land and IWVJ performance measures among elite male water-polo players by considering body density when dry-land performance measures were expressed. We demonstrated that measuring IWVJ height with the use of a linear encoder is highly reliable. We found that CMJ impulse but neither SJ impulse nor isometric squat force correlated with IWVJ height. Finally, body density seems to have a negligible contribution to IWVJ height, which is an important information when body composition management is in focus among elite male water polo players.
